# High Prevalence of Mucosa-Associated *E. coli* Producing Cyclomodulin and Genotoxin in Colon Cancer

**DOI:** 10.1371/journal.pone.0056964

**Published:** 2013-02-14

**Authors:** Emmanuel Buc, Damien Dubois, Pierre Sauvanet, Jennifer Raisch, Julien Delmas, Arlette Darfeuille-Michaud, Denis Pezet, Richard Bonnet

**Affiliations:** 1 UMR 1071 Inserm/Université d'Auvergne, Clermont Université, Clermont-Ferrand, France; 2 USC 2018, INRA, Clermont-Ferrand, France; 3 Service de Bactériologie, CHU Clermont-Ferrand, Clermont-Ferrand, France; 4 Service de Chirurgie digestive, CHU Clermont-Ferrand, Clermont-Ferrand, France; Louisiana State University and A & M College, United States of America

## Abstract

Some *Escherichia coli* strains produce toxins designated cyclomodulins (CMs) which interfere with the eukaryotic cell cycle of host cells, suggesting a possible link between these bacteria and cancers. There are relatively few data available concerning the colonization of colon tumors by cyclomodulin- and genotoxic-producing *E. coli*. We did a qualitative and phylogenetic analysis of mucosa-associated *E. coli* harboring cyclomodulin-encoding genes from 38 patients with colorectal cancer (CRC) and 31 with diverticulosis. The functionality of these genes was investigated on cell cultures and the genotoxic activity of strains devoid of known CM-encoding gene was investigated. Results showed a higher prevalence of B2 phylogroup *E. coli* harboring the colibatin-producing genes in biopsies of patients with CRC (55.3%) than in those of patients with diverticulosis (19.3%), (p<0.01). Likewise, a higher prevalence of B2 *E. coli* harboring the CNF1-encoding genes in biopsies of patients with CRC (39.5%) than in those of patients with diverticulosis (12.9%), (p = 0.01). Functional analysis revealed that the majority of these genes were functional. Analysis of the ability of *E. coli* to adhere to intestinal epithelial cells Int-407 indicated that highly adherent *E. coli* strains mostly belonged to A and D phylogroups, whatever the origin of the strains (CRC or diverticulosis), and that most *E. coli* strains belonging to B2 phylogroup displayed very low levels of adhesion. In addition, 27.6% (n = 21/76) *E. coli* strains devoid of known cyclomodulin-encoding genes induced DNA damage *in vitro*, as assessed by the comet assay. In contrast to cyclomodulin-producing *E. coli*, these strains mainly belonged to A or D *E. coli* phylogroups, and exhibited a non significant difference in the distribution of CRC and diverticulosis specimens (22% *versus* 32.5%, p = 0.91). In conclusion, cyclomodulin-producing *E. coli* belonging mostly to B2 phylogroup colonize the colonic mucosa of patients with CRC.

## Introduction

Colorectal cancer (CRC) is currently the third most common cancer in men and women, and the fourth leading cause of cancer death worldwide, with 1.2 million estimated cases and 609,000 estimated deaths per year [1]. Sporadic CRC accounts for about 95% of colorectal cases [2]. Genetic factors are usually thought to account for about 20% of cancer causation with environment contributing the remaining 80% risk [3]. CRC is therefore strongly associated with environmental exposures including bacteria that contribute significantly to the colonic environment. Evidence has accumulated showing that the composition of human intestinal microbiota influences host health status. Microbial dysbiosis is observed in CRC patients and evidence of bacterial interactions in CRC has been reported for *Streptococcus bovis*, *Enterococcus spp.*, *Helicobacter pylori*, *Bacteroides fragilis* and *Escherichia coli* (for review see [4]). Various studies clearly demonstrate a link between mucosally adherent *E. coli* and CRC. Studies of cancer patients in the UK and Germany revealed that mucosa-associated *E. coli* are more frequently identified in colon tissue from patients with adenocarcinomas than in that of controls [5], [6]. Swidsinski *et al.* reported that only 3% of colon mucosa biopsies from asymptomatic controls tested were positive for *E.* coli with a bacterial universal PCR [5]. In contrast, biopsies from 92% of patients with colonic adenomas or carcinomas harbored bacteria, with *E. coli* being predominant in 70% of patients [5]. Similarly, Martin *et al.* found that 70% of CRC patients had mucosa-associated bacteria, and that a significant proportion of bacteria belonged to the *E. coli* species. Interestingly, Bronowski *et al.* showed that some *E. coli* strains carry virulence genes previously categorized as specific to uropathogenic *E. coli* (UPEC) [7]. Such *E. coli* strains may trigger cellular proliferation in intestinal tract [8], as seen with other bacteria [9], [10].

*E. coli* is the predominant aero-anaerobic Gram-negative species of the normal intestinal flora and participates in promoting the stability of the luminal microbial flora and in maintaining normal intestinal homeostasis [11]. As a commensal, *E. coli* coexists with its mammalian host in good harmony and rarely causes disease. However, some strains carry a combination of virulence genes that enable them to cause intestinal (InPEC, Intestinal Pathogenic *E. coli*) and extra-intestinal (ExPEC, Extraintestinal Pathogenic *E. coli*) infections (for reviews see [12]–[14]). Phylogenetic analysis has shown that *E. coli* is composed of four main phylogenetic groups (A, B1, B2, and D)[15], [16]. Pathogenic strains belong mainly to groups B2 and D, while most fecal strains belong to groups A and B1. Strains of groups B2 and D often carry virulence factors that are lacking in group A and B1 strains [17]–[19].

Among *E. coli* virulence factors, several toxins, called cyclomodulins, are attracting growing attention because they are genotoxic and/or modulate cellular differentiation, apoptosis, and proliferation (for review see [4]). Cytotoxic necrotizing factor (CNF) activates Rho GTPases, which leads to cytoskeletal alterations and affects the cell cycle. The cycle-inhibiting factor (CIF) target NEDD8-conjugated cullins to hijack host-cell signaling pathways [20], [21]. The genotoxin colibactin is a hybrid polyketide-non ribosomal peptide compound [22]. Its biosynthesis machinery is encoded by the *pks* genomic island. Colibactin causes DNA double-strand breaks and a chromosomal instability in human eukaryotic cells [22], [23]. The cytolethal distending toxin (CDT) also induces DNA damage probably through DNAse activity, and a closely related enzyme produced by *Helicobacter hepaticus* promotes the progression of hepatitis to pre-malignant, dysplastic lesions and increases the proliferation of hepatocytes, providing the first evidence that CDT has carcinogenic potential *in vivo* [24]–[26].

In the present study, we compared the prevalence of cyclomodulin- and genotoxin-encoding genes in mucosa-associated *E. coli* strains from resection specimens of CRCs and diverticulosis. We also investigated the genotoxic activity of mucosa-associated *E. coli* devoid of known genotoxin-encoding genes and their ability to adhere to epithelial intestinal cells.

## Materials and Methods

### Ethics Statement

Ethical approval for the study was granted by the Clermont-Ferrand research ethics committee. This IRB allowed for the waiver of written consent and approved the process of obtaining verbal consents from potential subjects, because the research involves no procedures for which written consent is normally required outside of the research context and presents no risk of harm to subjects. The biological samples were collected from colon resections, which were required for the treatment of patients. The investigators explained the study to the potential subject verbally, providing all pertinent information such as purpose, procedures, putative risks. Following this verbal explanation, the potential subject was provided with a study information sheet. After allowing the potential subject time to read the study information sheet, the investigator answered any additional questions the potential subject may have had. A verbal agreement to participate in the research was obtained for all patients included in the study. The dates of verbal consent were tracked in a non-identifiable manner.

### Patients

In order to study macroscopic samples from resection of colon specimens, we compared patients with CRC and patients with diverticulosis as a non-cancer group. Sixty-nine patients were studied between March 2007 and November 2009 at the university hospital of Clermont-Ferrand, France. Thirty-eight had CRC, and 31 had complicated diverticulosis (Tables S1, S2, S3). Patients from the CRC group had uncomplicated and resectable colon cancer developed either in the proximal colon (from the cecum to the hepatic flexure of the ascendant colon) or in the distal colon (sigmoid colon). CRC of the transverse or the descendant colon were excluded because of their low occurrence, and to avoid the risks of bias. In all cases, the operation consisted in segmental resection of the colon involved by the tumor with immediate anastomosis without diverting stoma. Patients with complicated CRC (obstruction, perforation or infection) were excluded from the study. Patients from the diverticulosis group had diverticulosis involving the sigmoid colon and required surgery because of a history of complication (recurrent diverticulitis, abcess, peritonitis). We excluded patients with acute or chronic inflammation at the time of surgery, and those with stenosis. In the event of a recent attack of diverticulitis, antibiotics were stopped at least 3 weeks before surgery. Sex ratio was 1.05 and 0.72 for CRC and DIV patients respectively. The age range was 35–95 years for cancer patients (median age, 71 years and average age, 67 years) and 34–81 years for diverticulosis patients (median age, 58 years and average age, 60 years). Samples were taken on resected colon, at the site of malignant tumors for CRC patients and in normal mucosa for diverticulosis patients. Pathologic analysis confirmed the neoplastic features of the samples in CRC patients, and the lack of inflammation or dysplasia in diverticulosis patients. The CRC series comprised 21 proximal and 17 distal colon samples. TNM stage is reported in Tables S1 and S2. Bowel preparation was by oral sodium picosulfate or oral polyethylene glycol the evening before surgery. All resection patients had received cefoxitin (2 g intravenously) at the time of incision and none had received antibiotics in the 4 weeks before sampling.

### Sample treatment

The mucosal samples were placed in 10 mL of sterile phosphate buffered saline pH 7.4 (PBS) and transported on ice to the laboratory. The samples were weighed (50 to 100 mg each) and washed thoroughly three times in 10 mL PBS to remove most of the fecal bacteria. Each washing step was followed by centrifugation at 900g for 5 minutes. The specimens were then crushed (Ultra-Turrax, IKA) and incubated for 15 minutes on a tube rotator at room temperature in the presence of Triton 0.1X. Tenfold dilutions of the lysate were then plated on Drigalski agar and chromogenic agar chromID CPS3® (bioMérieux), which allow the identification of *E. coli*. *E. coli* colonies were collected after 24 hours of incubation at 37 °C and the identification of bacteria was confirmed with the automated Vitek II® (bioMérieux) system. When possible a maximum of 96 *E. coli* colonies per sample were collected for molecular typing. The bacteria were subcultured for 24 hours at 37 °C in 96-well plates in Luria Bertani medium, supplemented with 15% glycerol and then stored at −80 °C.

### Molecular typing and phylogenetic grouping

Ten colonies per sample were typed with molecular methods to identify the *E. coli* strains (*E. coli* genotypes) colonizing the samples. Two genotyping methods were used, an “Enterobacterial Repetitive Intergenic Consensus” sequence (ERIC)-PCR using primer ERIC2 (5′-AAG TAA GTG ACT GGG GTG AGC G-3 ′) and a “Random Amplified Polymorphic DNA” (RAPD)-PCR using primer 1283 (5′-GCG ATC CCC A-3 ′) [27], [28]. One representative strain was subsequently analysed and stored at -80 °C in Luria-Bertani medium supplemented with 15% glycerol. *E. coli* strains were then classified according to the *E. coli* Reference Collection (ECOR) system [16] into phylogenetic groups A, B1, B2, and D using a multiplex PCR technique [29]. Strain RS218, which harbors all the genes targeted by the multiplex PCR, was used as positive control.

### Detection and identification of cyclomodulin-producing genes

Cyclomodulin-encoding genes were detected by dot-blot DNA hybridization experiments in all *E. coli* strains. The probes were obtained by PCR as previously described [30] (Tables S4 and S5) using the PCR DIG probe synthesis kit (Roche Applied Sciences, Switserland) according to the manufacturer's instructions. Two-microgram DNA samples were fixed onto positively charged nylon membranes by UV illumination for 20 min. Hybridization was performed with the Roche labeling and detection kit (Roche Applied Sciences) as indicated by the manufacturer. Each spot was checked with a 16S rRNA gene probe. The *pks* island, which contains the colibactin-producing gene cluster, was screened with a probe overlapping the *clbK* and *clbJ* genes. The *cnf* genes were detected with a mixture of probes specific to *cnf1*, *cnf2*, and *cnf3*. The *cdtB* genes were detected by two hybridization experiments with the *cdtB-II-cdtB-III-cdtBV* and *cdtB-I-cdtB-IV* probe mixtures. The *cif* gene was detected using an internal specific probe. The sensitivities and specificities of the probes were checked on each membrane by spotting DNA extracts of all cylomodulin control strains. Positive hybridizations with a cyclomodulin probe were subjected to confirmatory PCR assays as previously reported [30]. The reaction mixture contained 50 ng DNA sample, 0.2 mM each deoxynucleoside triphosphate (dNTP), 0.4 µM each primer, 3 mM MgCl2, and 1.0 U RedGoldStar DNA polymerase (Eurogentec, Belgium) in the corresponding reaction buffer. Primers located in the 5′ and 3′ regions of the pks island (the clbA and clbQ genes) were used to confirm the full presence of the colibactin-producing island.

### Cytopathic assays

HeLa cells (derived from cervical cancer) purchased from ATCC (ATCC® CCL-2^TM^) were maintained in an atmosphere containing 5% CO2 at 37 °C in appropriate medium. The cells were cultured in DMEM medium supplemented with 10% (vol/vol) fetal calf serum (Lonza, Walkersville, MD USA), 1% L-glutamine (Life-Technologies), 200U of penicillin, 50 mg of streptomycin, 0.25 mg of amphoterocin B per liter, and 1% hepes buffered saline solution (Lonza). They were seeded in 96-well tissue culture plates at 5×10^4^ cells/well for 24 H. The cytopathic effects of CNF and CDTs were investigated in all strains from cell-lysate, as previously described [31]. Briefly, the effects of CDT and CNF were detected by a cell-lysate-interacting test. After 48 H culture at 37 °C with shaking in Luria-Bertani broth medium, bacterial cells were sonicated and sterile filtered separately using 0.22-µm-pore-size filters. HeLa cells were treated with the sterile sonicated lysates (final protein concentration: 4 µg/ml) until analysis. The effects of colibactin and Cif were detected by a cell-bacterium-interacting test, based on the interaction between HeLa cells and bacteria. Overnight Luria-Bertani broth cultures of *E. coli* were washed three times and diluted in interaction medium. HeLa cell cultures were infected at multiplicities of infection (MOI, numbers of bacteria per cell at the onset of infection) of 100 and 200. The cells were washed 4 H after inoculation and incubated in cell culture medium with 200 µg/ml gentamicin until analysis. After 72 H of incubation at 37 °C under a 5% CO2 atmosphere, the medium was removed by three washes of the HeLa cell monolayers. The morphological changes characteristic of CDT, CNF, colibactin, and Cif were observed after staining with Giemsa stain. Identification was based on the ability of either bacterial lysate containing CNF and CDT or whole live bacteria producing colibatin and CIF to induce a cytopathic effect on epithelial cells analyzed at 3-days post infection. Colibactin, CDT and CIF induced cytopathic effects, as evidenced by enlarged nuclei and cell distension (megalocytosis), while CNF induced multinucleation, and enlargement of HeLa cells. Multinucleation is observed in >50% of cells infected by CNF-producing bacteria and in around 10% of non-infected cells or cells infected with other CM-producing strains. For CNF and CDT, the cytopathic effect is only observable with bacterial lysates. In contrast, for colibactin and CIF, contact between bacteria and host cells is required. The detection of alpha-hemolysin was performed for all strains studied by overnight growth at 37 °C on Columbia sheep blood (5%) agar (Oxoid, Dardilly, France). *E. coli* 25922 (ATCC) was used as the reference strain for alpha-hemolysin production.

### Single-cell gel electrophoresis

The genotoxic activity of *E. coli* strains devoid of known CM-encoding genes were investigated using single-cell gel electrophoresis (comet assay). HeLa cell cultures were infected at MOI of 500 with *E. coli* cultured overnight in Luria-Bertani broth. The cells were washed twice 3 H after inoculation and were incubated overnight in cell culture medium with 200 µg/ml gentamicin at 37 °C under a 5% CO_2_ atmosphere. They were then washed with PBS medium and combined with 0.5% low-melting-point agarose (Bio-Rad, Marnes La Coquette, France) dissolved in sterile PBS at 37 °C. The cell-agarose mixture was applied to a microscope slide precoated with 1.5% normal-melting-point agarose (Molecular Biology Grade, Bio-Rad) dissolved in sterile PBS at 37 °C. A cover slip was applied and allowed to solidify at 4 °C for 60 min. Slides were then placed in 50 mL lysis buffer (10 mM Tris-HCl, 2.5 M NaCl, 100 mM EDTA containing 1% Triton X-100, 10% DMSO, pH 10) for 2 h at 4 °C in the dark. Slides were immersed in electrophoresis buffer (1 mM EDTA 300 mM NaOH pH 13) for 1 h at 4 °C and an electric field was applied (1 V/cm) for 40 minutes. The slides were neutralized with 400 mM Tris-HCl pH 7.5 and dried. 40 µl of a 1:10,000 dilution of SybrGreen was applied directly to the slide. Individual cells or comets were viewed by Zeiss Axioplan2 fluorescence microscope. The B2 *E. coli* strain IHE3034 and *E. coli* DH10β pBACpks were used as positive controls [22]. The B2 *E. coli* strain IHE3034 Δ*clbP* and *E. coli* DH10β pBAC were used as negative controls [22].

### Adhesion assay

Int-407 cells (derived from human intestinal embryonic jejunum and ileum) purchased from ATCC (ATCC® CCL-6^TM^) were maintained in an atmosphere containing 5% CO2 at 37 °C in appropriate medium. They were cultured in DMEM medium supplemented with 10% (vol/vol) fetal calf serum (Lonza, Walkersville, MD USA), 1% L-glutamine (Life-Technologies), 200U of penicillin, 50 mg of streptomycin, 0.25 mg of amphoterocin B per Liter, and 1% hepes buffered saline solution (Lonza). Briefly, cells were seeded at a density of 2×10^5^ cells/cm2 in culture plates for 48 H. The infection was performed at a multiplicity of infection of 10 bacteria per cell. Infected cells were centrifuged at 900 g for 10 min at 25 C and placed at 37 °C for 3 H. Cells were washed three times in phosphate-buffered saline (PBS; pH 7.2). The epithelial cells were then lysed with 1% Triton X-100 (Sigma) in deionised water. Samples were diluted and plated onto Luria-Bertani (LB) agar plates to determine the number of CFU corresponding to the total number of cell-associated bacteria. Strains *E. coli* K12 and *E. coli* LF82 were used as negative and positive controls, respectively [32]. The results are expressed as number of adherent bacteria per cell after a 3 H infection period.

### Statistical analysis

Statistical analysis was performed using the Fisher exact and chi-square tests. For multiple-group comparisons, an initial chi-square test for heterogeneity was done, and only if this yielded a P value of <0.05 were the individual pairwise comparisons tested.

## Results

### *E. coli* strains in colon cancer and diverticulosis samples

The analysis of *E. coli* strains indicated that the number of samples without *E. coli* was significantly higher in diverticulosis patients (19.4%, n = 6/31) than in those with CRC (2.6%, n = 1/38), p = 0.04 (Table 1). Many colonic specimens harbored only one *E. coli* genotype. This was observed in 42.1% (n = 16/38) of CRC specimens and in only 25.8% (n = 8/31) of diverticulosis but the difference was not significant (p = 0.12). Most strains isolated from CRC belonged to the B2 phylogroup (73.7%, n = 28/38) in contrast to those isolated from diverticulosis (41.9%, n = 13/31), p<0.01 (Table 2). No significant difference in *E. coli* phylogroup distribution was observed, including for B2 *E. coli* strains isolated from proximal (82.4%, n = 14/17) and distal colon tumors (66.7%, n = 14/21), p = 0.23 (Table 2). Overall *E. coli* strains belonging to the B2 phylogroup colonized colon tumors more frequently than they did diverticulosis samples.

**Table 1:** Number of *E. coli* strains collected from patients with CRC and diverticulosis.

	Percentage (number) of samples containing *E. coli* strains			
Number of *E. coli* strains per sample	Diverticulosis (n = 31)	CRC (n = 38)	Proximal colon cancer (n = 21)	Distal colon cancer (n = 17)
0	19.4 (6)	2. 6 (1)	4.8 (1)	0.0 (0)
1	25.8 (8)	42.1 (16)	47.6 (10)	35.2 (6)
>1	54.8 (17)	55.3 (21)	47.6 (10)	64.7 (11)

**Table 2:** Colonization of diverticulosis and CRC samples by *E. coli* phylogroups (A, B1, B2 and D).

	Percentage (number) of samples containing *E. coli*			
	A	B1	B2	D
Diverticulosis (n = 31)	41.9 (13)	6.5 (2)	41.9 (13)	32.2 (10)
CRC (n = 38)	28.9 (11)	23.7 (9)	73.7 (28)	26.3 (10)
Distal colon cancer (n = 17)	29.4 (5)	17.6 (3)	82.3 (14)	41.1 (7)
Proximal colon cancer (n = 21)	28.5 (6)	28.6 (6)	66.7 (14)	14.3 (3)

### Distribution of CM-encoding genes according to *E. coli* phylogenetic groups

The distribution of CM-encoding genes in *E. coli* strains is shown in Table 3 and Tables S1, S2, S3. The most frequent trait was the colibactin-encoding *pks* island (80% of CM-producing *E. coli*, n = 28/35 and 24.1% of total *E. coli* strains, n = 28/116). All strains associated with the colonic mucosa of patients with CRC or diverticulosis harboring the colibactin-encoding *pks* island belonged to phylogroup B2 (p <0.001 for group B2 *versus* groups A, B1 and D, individual or combined). In addition, 16.4% (n = 19/116) of strains possessed the *cnf* gene; of these only one was *cnf2*-positive and none was *cnf3*-positive, indicating that these strains were not of animal origin [33], [34]. All *cnf1*-harboring strains belonged also to phylogroup B2, and accounted for 36.7% (n = 18/49) of strains of this phylogroup. As for the *pks* island, the association *cnf1* with phylogroup B2 was strong (p<0.001 for group B2 *versus* groups A, B1 and D, individual or combined), and 33% (n = 16/49) of B2 strains possessed both the *pks* island and the *cnf1* gene (p<0.001 for group B2 versus groups A, B1 and D, individual or combined), as previously observed [30], [35]. All but three strains harboring *cnf1* gene exhibited the alpha-hemolytic phenotype. However, the three non-hemolytic *cnf1*-positive strains harbored the *hlyC* gene. The *cdtB* genes were observed in six strains. Although four out of six *cdt*-positive strains belonged to phylogroup B2, no significant association with a particular phylogenetic group was observed, even with the different *cdtB* gene subtypes. *cdtB-I*/*cdtB-IV* genes (n = 5) were more frequently observed than those of the *cdtB-II-cdtB-III-cdtBV* gene group (n = 1). The only *cdt-III*-positive strain also harbored the *cnf2* gene, a combination of genes frequently reported in the pVir plasmid and mainly seen in strains of bovine origin [36], [37]. The *cdtB* genes showed no particular association with the colibactin-encoding *pks* island or the *cnf1* gene. Since *cdt* genes have been extensively studied in Shiga toxin-producing *E. coli* (STEC) strains [38]–[40], it was decided to investigate *cdtB*-positive strains for *stx* and *eae* genes. No *stx* or *eae* gene was detected in *cdtB*-positive strains. The *cif* gene was detected in three strains that belonged to phylogroups A (n = 1) and B1 (n = 2) and harbored no other CM-encoding genes. CIF is an effector of the type 3 secretion system encoded by the locus of enterocyte effacement (LEE) observed in enteropathogenic *E. coli* (EPEC) and enterohemorrhagic *E. coli* (EHEC) [41], [42] and the three positive strains in this study possessed the *eae* gene but neither *stx1* nor *stx2* genes, and therefore belonged to the EPEC pathotype.

**Table 3:** Distribution of CM-encoding genes in *E. coli* strains according to phylogroups (A, B1, B2 and D).

	Percentage (number) of *E. coli* belonging to the phylogroup				
	A (n = 33)	B1 (n = 12)	B2 (n = 49)	D (n = 22)	All (n = 116)
*pks*	0.0 (0)	0.0 (0)	57.1 (28)	0.0 (0)	24.1 (28)
*cnf*	0.0 (0)	8.3 (1)	36.7 (18)	0.0 (0)	16.4 (19)
*cdt*	3.0 (1)	8.3 (1)	8.2 (4)	0.0 (0)	5.2 (6)
*cif*	6.1 (2)	8.3 (1)	0.0 (0)	0.0 (0)	2.6 (3)
*cm* 1	9.1 (3)	16.7 (2)^2^	63.3 (31)^2^	0.0 (0)	30.2 (36)^2^

1 , cyclomodulin-encoding gene;

2 , some *E. coli* strains harbored more than one CM-encoding genes.

### Phenotypic detection of cyclomodulins

**Figure 1:**
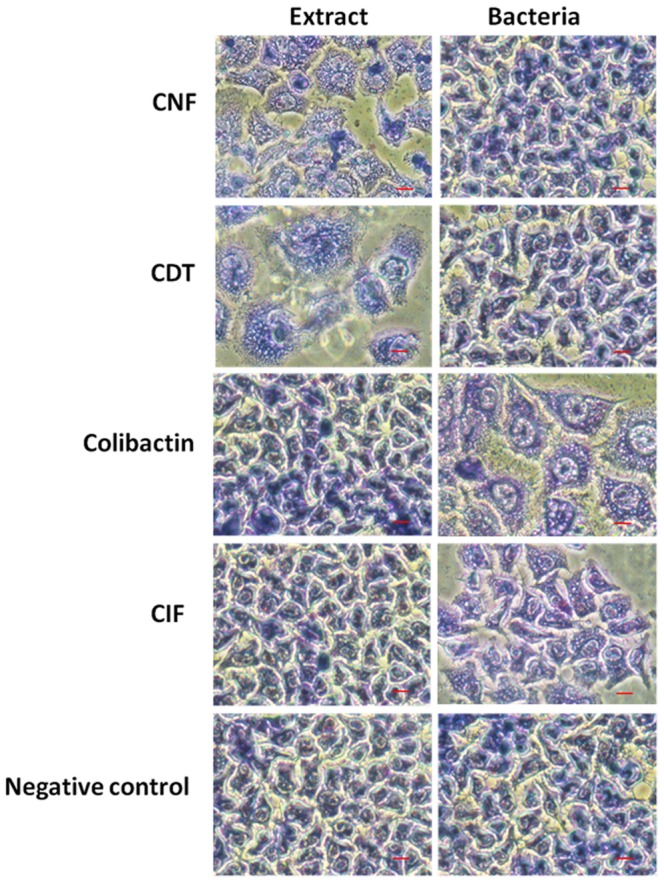
Cytopathic effect induced by *E. coli* live bacteria or protein extracts on epithelial cells at three-day post-infection. For CNF and CDT, the cytopathic effect is only observable with bacterial lysates. In contrast, for colibactin and CIF, a contact between bacteria and host cells is required. Colibactin, CDT and CIF induced cytopathic effects as seen by enlarged nuclei and cell distension (megalocytosis), while CNF induced multinucleation and enlargement of HeLa cells.

Cell-lysate-interacting tests were used to investigate CNF and CDT production in all strains. The detection of colibactin and CIF production, which require cell-bacterium-interacting tests, was only possible in the non-hemolytic strains, because the hemolysin-producing strains, in contrast to the corresponding lysates, induced rapid cell death. The results are given in Tables S1, S2, S3. Colibactin, CDT and CIF induced cytopathic effects, as evidenced by enlarged nuclei and cell distension (megalocytosis), while CNF induced multinucleation in ≥50% of cells and enlargement of HeLa cells (Figure 1). For CNF and CDT, the cytopathic effect was only observable with bacterial lysates, whereas a contact between bacteria and host cells was required for colibactin and CIF, as previously reported (Figure 1) [22], [31], [42]. The observation of a cytopathic effect was associated to the presence of CM-encoding genes. Three strains harboring a *pks* island isolated from diverticulosis, distal and proximal colon specimens did not induce any cytopathic effect. One strain isolated from a diverticulosis sample had a nonfunctional *cnf1* gene and two *cdt*-positive strains showed no cytopathic effect. For the strain harboring *cnf2* and *cdt*-III genes, >50% cell multinucleation attested to the production of CNF and the wide megalocytosis attributed to CNF production or its combination with CDT-III. A cytopathic effect was observed for the strains harboring the *cif* gene. Overall, most CM-encoding genes were functional, notably *cnf* and *cif* (93% and 100%, respectively).

### Distribution of CM-encoding genes according to the specimen origin and the phylogroupe

**Figure 2:**
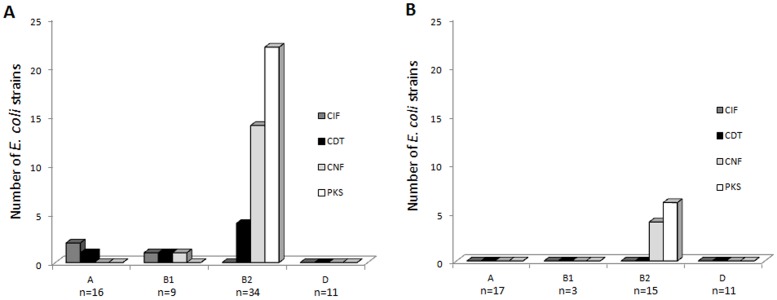
Distribution of *E. coli* strains producing various cyclomodulins according to phylogroups and specimen origins. A, *E. coli* strains (n = 70) isolated from CRC samples (n = 38), and B, *E. coli* strains (n = 46) isolated from diverticulosis samples (n = 31).

The results are shown in Table 4, Figure 2 and Tables S1, S2, S3. Twenty-five CRC specimens among 38 (65.8%) contained CM-positive *E. coli* and only 6 diverticulosis specimens among 31 (19.4%), indicating that CM-harboring strains were preferentially associated with CRC (p<0.01). This difference was associated with a high number of CM-positive B2 *E. coli* in CRC specimens compared to that observed in diverticulosis specimens (Figure 2). Accordingly, strains harboring colibactin-encoding *pks* island were present in 55.3% of CRC specimens (n = 21/38) and only in 19.3% of diverticulosis specimens (n = 6/31), indicating that *pks* positive strains were significantly (p<0.01) more prevalent in CRC. To a lesser extent, *cnf* and *cdt* positive *E. coli* were significantly (p≤0.02) more prevalent in the CRC than in the diverticulosis samples. These differences were mainly due to the high prevalence of *pks*-, *cnf*- and *cdt*-positive *E. coli* in distal CRC specimens compared to that in diverticulosis specimens (p≤0.01). Altogether, these results indicate that CM-positive *E. coli* distribution differs according to specimen origin.

**Table 4:** CM-encoding gene content of CRC and diverticulosis samples.

	Number (percentage) of specimens exhibiting CM-encoding genes					
	*pks*	*cnf*	*cdt*	*cif*	*cm* 1,2	
Diverticulosis (n = 31)	6 (19.3)	4 (12.9)	0 (0.0)	0 (0.0)	6 (19.4)	
CRC (n = 38)	21 (55.3)	15 (39.5)	6 (15.8)	3 (7.9)	25 (65.8)	
Distal colon cancer (n = 17)	11 (64.7)	9 (52.9)	4 (23.5)	1 (5.9)	13 (76.5)	
Proximal colon cancer (n = 21)	10 (47.8)	6 (28.6)	2 (9.5)	2 (9.5)	12 (57.1)	

1 , cyclomodulin-encoding gene;

2 , some *E. coli* strains harbored more than one CM-encoding genes.

### Genotoxicity of *E. coli* devoid of CM-encoding genes

**Figure 3:**
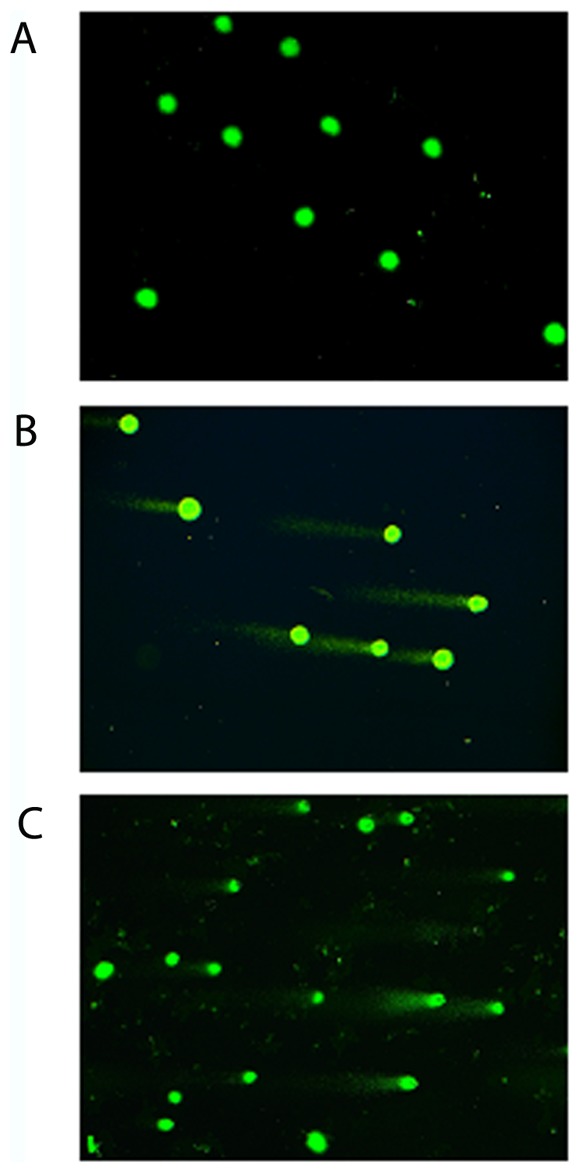
DNA damage was detected by the comet assay in HeLa cells exposed to *E. coli* for 3 h. DNA damage was not detected in HeLa cells infected with the *E. coli* strain IHE3034 Δ*clbP* harboring a defective *pks* island (A). Comet assay was positive in HeLa cells infected with the *E. coli* strain IHE3034 harboring *pks* island (B) or with *E. coli* devoid of known cyclomodulin-encoding genes (C).

We investigated whether *E. coli* devoid of known CM-encoding genes can induce DNA damage in host cells. Using HeLa cultured cells, the genotoxicity of strains was investigated by single-cell gel electrophoresis assay (or comet assay), the state-of-the-art technique for detecting DNA damage caused by chemical genotoxins. A total of 76 clinical *E. coli* strains were investigated; 15 originated from proximal colon cancer, 21 from distal colon cancer and 40 from diverticulosis. These strains belonged to A (n = 29), D (n = 21) and B2 (n = 17) phylogroups. Interestingly, 27.6% (n = 21/76) of *E. coli* strains devoid of known CM-encoding genes induced the formation of comets, which are typical of host cell DNA damage (Figure 3 and Tables S1, S2, S3). In addition, in contrast to CM-producing *E. coli* strains, which belong mainly to the B2 phylogroup, these comet positive strains belonged mainly to A (52%, n = 11/21) and D (29%, n = 6/21) phylogroups. Comets were observed with 13 strains (32.5%) among 40 isolated from patients with diverticulosis, and with 8 strains (22%) among 36 in patients with CRC. The distribution of these putative genotoxic strains in the specimens was therefore different from that of CM-encoding strains.

### Bacterial adhesion to intestinal epithelial cells

**Figure 4:**
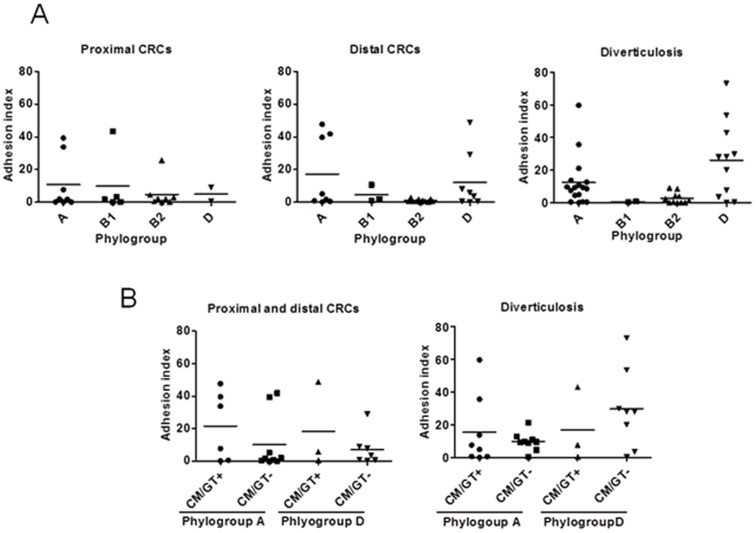
Adhesion ability of *E. coli* strains isolated from diverticulosis and CRC (proximal and distal) samples to Int-407 intestinal epithelial cells. A, adhesion ability according to the specimen origin and E. coli phylogroup. B, Adhesion ability of *E. coli* strains isolated from diverticulosis and CRC (proximal and distal) samples and belonging to A and D phylogroups according to their ability to produce or not a cyclomodulin/genotoxin. The results are expressed as number of adherent bacteria per cell after 3 h infection period. Data are means+/−SEM for at least 3 independent experiments.

The ability of *E. coli* strains to adhere to Int-407 epithelial cells was compared according to the origin of the strains, their phylogroup and their ability to produce cyclomodulin(s) or unknown genotoxin (positive comet assay). As shown in Figures 4A and B, highly adherent *E. coli* strains mostly belonged to A and D phylogroups, irrespective of the origin of the strains (CRC or diverticulosis). Unexpectedly, most *E. coli* strains belonging to B2 phylogroup displayed very low levels of adhesion. Analysis of the adhesive properties of A and D *E. coli* strains according to their ability to produce cyclomodulin(s) or unknown genotoxin (positive comet assay) showed that there was no significant difference (Figure 4B).

## Discussion

The study of colonic mucosa-associated *E. coli* from patients with CRC or diverticulosis indicated that (i) *E. coli* strains belonging to the B2 phylogroup colonized colon cancers more frequently than they did diverticulosis samples, (ii) the CM-encoding genes were overrepresented in colon cancers, especially colibactin-encoding *pks* island, *cnf1* and *cdt* gene, and (iii) the distal colon cancers were more frequently colonized by B2 *E. coli* producing CMs than were the diverticulosis samples.

The *E. coli* strains of the B2 phylogroup are mostly involved in extra-intestinal infections, such as urinary tract infections. They produce numerous virulence factors, notably pili adhesins, that favor colonization of the urinary tract. We can speculate that such adhesins specifically found in B2 *E. coli* also favor the colonization of colon cancer. Bronowski *et al.* observed UPEC-associated genes encoding adhesins among a panel of mucosa-associated *E. coli* isolated from colon cancer, and Martin et *al* reported that *E. coli* strains isolated from colorectal tumors frequently expressed hemagglutinins, which favor adhesion to intestinal epithelial Int-407 and HT29 cells [7], [43]. One explanation of the high prevalence of B2 *E. coli* in CRC could be that changes in the host mucosa receptor repertoire have an effect on the bacterial population associated with mucosa. A higher level of colonization with B2 *E. coli* has also been observed in inflammatory bowel disease (IBD) [44]. In Crohn's disease patients, such colonization was accompanied by increased ileal expression of the glycoprotein CEACAM6, which acts as a receptor for type 1 pili produced by *E. coli* [45], [46]. Interestingly, Crohn's disease-associated B2 *E. coli* strains can induce the expression of CEACAM6 in intestinal epithelial cells, and yet CEACAM6 is a human tumor maker, whose overexpression has been observed in colonic tumors [47], [48].

In this study, the CM-encoding genes were overrepresented in CRC compared with diverticulosis samples. Similar results were obtained by Arthur *et al.* when comparing the prevalence of colibactin-producing *E. coli* in CRC patients compared to control patients with diverticulosis, sporadic polyposis, irritable bowel syndrome or hemorrhoids [49]. Our data, which showed differences in prevalence of CM-producing *E. coli* strains belonging to B2 phylogroup, may be underestimated due to our experimental procedure of preparing colonic specimens. We removed most fecal bacteria by washing the specimens thoroughly three times in PBS, but some mucus-associated bacteria were still present. Two studies, by Swidsinski *et al.* and Martin *et al.*, reported higher numbers of *E. coli* in colon cancer samples than in controls when biopsy samples were studied after surface mucus removal, indicating that higher numbesr of *E. coli* are in very close association with the mucosa in CRC samples compared to those of controls. The CM-encoding genes identified were *pks* island, *cnf1* and *cdt* gene. Accordingly, Bronowski *et al.* have previously identified among a panel of 10 *E. coli* isolated from biopsies of colon cancers, 3 strains harboring *cnf1* genes and 4 strains harboring a polyketide synthase-encoding gene belonging to *pks* island [7]. The high prevalence of CM-encoding genes in CRC suggests a possible role of CM-encoding *E. coli* in the development of malignant colon tumors. It is well established that colibactin-encoding *pks*-harboring *E. coli* strains are mutagenic and genotoxic *in vitro* and *in vivo* [22], [23]. They can promote CRC without affecting intestinal inflammation [50]. In addition, transient infection of human cell lines by such strains induces anchorage-independent colony formation [23], a process involved in metastases. Because of these mutagenic and cell transformation activities, *E. coli* strains harboring the colibactin-encoding *pks* island may affect carcinogenesis at different stages. Cdt also induces DNA breaks in eukaryotic cells [26]. The presence of bacteria harboring *cdt* have already been associated with some lymphomas of the small intestine and can promote the development of hepatic and colon tumors [25], [51]–[54]. CNF promotes cell proliferation by activating the Rho-GTPases and stimulates the transition from G_1_ to S phases [55]. In addition, CNF1 inhibits apoptosis and alters tight junction structure and epithelial barrier function [56]–[58], processes that could favor carcinogenesis. Maddocks *et al.* have shown that EPEC are more frequent in the colon adenocarcinoma tissues than in matched normal colon tissues, with a prevalence of 25% and 0%, respectively [59]. EPEC inject into host cells bacterial effectors, which have an effect on DNA damage repair and the cytoskeleton [59], [60]. One effector is the product of the *cif* gene, which alters the ubiquitination process and thus the degradation of proteins involved in many cellular processes such as cell cycle regulation and cytoskeleton [21], [61]. In addition, the murine EPEC-like pathogen, *Citrobacter rodentium*, which harbors the LEE locus, is the cause of transmissible colonic hyperplasia [62], reduces the latency period of chemically induced tumors [63] and promotes colonic adenoma formation in APC/Min mice [64].

The distal colon cancers were more frequently colonized by B2 *E. coli* encoding CMs than the proximal colon cancers or diverticulosis samples, suggesting that the production of CMs directly or indirectly can provide a selective advantage for the colonization of distal colon cancers. This finding could be explained by major differences in the embryologic origin, bacterial flora composition, and physiology of the distal and proximal colons. The proximal colon derives from the midgut whereas the distal colon develops from the hindgut. The epithelial metabolism in the distal colon mainly involves butyrate, and in the proximal epithelium, acetate. Bacterial populations are qualitatively and quantitatively different in the lumen of proximal and distal colons [65], but studies showed that in a given patient similar populations of mucosa-associated bacteria are accommodated along the different parts of the colon irrespective of differences in the luminal content [66]–[68]. Most CM-producing strains harbor colibactin-encoding *pks* island or the combination of *cnf* genes with *pks* island (79% of CM-producing *E. coli*), suggesting an important role of *pks* island in the distribution of CM-producing *E. coli*. Nougayrède *et al.* proposed that the presence of colibactin-encoding *pks* can favor the colonization of the intestinal tract [22]. As a PK-NRP-type compound, colibactin requires specific precursors belonging to the bacterial secondary metabolism. The physiological features of the distal and proximal colons could modify *E. coli* metabolism and therefore the synthesis of PK-NRP compounds such as colibactin. Consequently, the efficiency of colibactin in promoting colonization may be affected and play a role in the distribution of CM-producing *E. coli*.

Interestingly, the proximal and distal colons also differ by certain aspects of their molecular physiopathology. The genomic instability associated with the microsatellite instability (MSI) phenotype is a hallmark of proximal colon cancer [69]. In contrast, chromosomal instability (CIN) is a major feature of distal colon cancers [69]. The *pks*-harboring *E. coli* induce major chromosomal damage and genetic instability [23]. Moreover, the CNF1-encoding gene, which is frequently associated with *pks* island, induces abnormal chromosome segregation. These CM-encoding *E. coli* strains may therefore have an impact on the development of colon cancers.

The B2 phylogenetic group of *E. coli* is attracting attention because it contains strains responsible for severe infections, and their genetic background is adapted to the acquisition and/or maintenance of numerous virulence factors [19]. However, the *E. coli* strains belonging to the A and D phylogroups account for a large proportion (40% to 55%) of the *E. coli* strains in the intestinal tract [17], [70], [71]. Surprisingly, we observed that *E. coli* strains belonging to A and D phylogroups, irrespective of the origin of the strains (CRC or diverticulosis), were highly adherent to intestinal epithelial cells compared to B2 *E. coli* strains, which displayed very low levels of adhesion. In addition, some *E. coli* strains belonging to A and D phylogroups in addition to having adhesion ability were able to induce DNA damage, suggesting that these strains have acquired unknown genotoxin(s). The distribution of these strains in CRC and diverticulosis specimens was not significantly different. The genotoxic *E. coli* strains of A and D subgroups, in contrast to B2 CM- producing *E. coli*, are therefore not specifically associated with CRC. However, their presence in close contact with colon tumors may still have an influence on the evolution of CRC.

The mechanism by which CM-producing *E. coli* strains can promote carcinogenesis is probably not due to the bacteria alone, and very likely involves numerous factors such as host susceptibility. DNA repair plays a pivotal role in maintaining genomic integrity with over 130 genes involved in various repair pathways that include base excision repair, nucleotide excision repair, double strand break repair and DNA mismatch repair [72]. In addition, polymorphisms within repair process genes are widely reported to be associated with an extensive range of malignancies that include CRC [73]–[75]. These polymorphisms can decrease their efficiency and explain susceptibility to the repeated aggressions of host DNA by resident *E. coli* producing genotoxins.

Finally, on the assumption that bacteria are associated with CRC, bacterial strains present at the origin of the cancer may disappear and be replaced by other bacteria better adapted to the cancer environment. In consequence, it remains difficult to determine whether the increase in specific bacteria is the consequence of the presence of malignant tissues or the cause of the cancer. However, these CM-producing bacteria, which colonize the malignant tumors, probably have an impact on the evolution of CRC.

In conclusion, our study showed a high prevalence of CM-producing B2 *E. coli* in biopsies of colon cancers, especially at the distal part. It suggests therefore a possible role of CM-producing *E. coli* in colon cancers. This could be investigated in a longitudinal observational study to clarify the role of CMs in colorectal carcinogenesis. It also emerged that the different endogenous features of the proximal and distal colons and their different responsiveness to exogenous factors probably lead to the emergence of specific bacterial populations which can affect carcinogenesis. As previously reported for ExPEC, cyclomodulins are mostly found in *E. coli* of the B2 phylogroup. However, we observed that colonic mucosa-associated *E. coli* belonging to the phylogroups A and D and devoid of known CM exhibit host cell genotoxic activity and should be considered as potentially harmful.

## Supporting Information

Table S1 ***E. coli*** **strains isolated from distal colonic cancers.**
(DOCX)

Table S2 ***E. coli*** **strains isolated from proximal colon cancers.**
(DOCX)

Table S3 ***E. coli*** **strains isolated from diverticulosis.**
(DOCX)

Table S4 **Primers used in this study.**
(DOCX)

Table S5 **Archetypal E. coli control strains used in this study.**
(DOCX)
