# Supplementary material for: High Prevalence of Mucosa-Associated E. coli Producing Cyclomodulin and Genotoxin in Colon Cancer
Source: PLoS One. 2013 Feb 14;8(2):e56964. doi: 10.1371/journal.pone.0056964 (PMC3572998; doi:10.1371/journal.pone.0056964)
Supplement: Table S1 — E. coli strains isolated from distal colonic cancers. (DOCX) [file pone.0056964.s001.docx]

**Table S1. *E. coli* strains isolated from distal colonic cancers.**

| Patient | *TNM stage^1^* | *E. coli* Strain | Phylogroup | Cytopathic effect | | | | CM-encoding gene | | | | Number of adherent  bacteria per cell |
| --- | --- | --- | --- | --- | --- | --- | --- | --- | --- | --- | --- | --- |
|  |  |  |  | Hly | pks-cif | cdt-cnf | comet | *pks* | *cnf* | *cdt* | *cif* |  |
| 1 | III | CFF1-7G2 | B2 | + | NA^2^ | + | NA^2^ | + | *cnf1* | - | - | NA^2^ |
|  |  | CFF1-7G1 | B2 | - | - | - | - | - | - | - | - | 0.5 |
|  |  | CFF1-7G4 | A | - | - | - | - | - | - | - | - | 5.1 |
| 3 | II | CFF3-1F8 | B2 | - | - | - | - | - | - | - | - | 2.0 |
| 8 | III | CFF8-2D5 | B2 | - | - | - | - | - | - | - | - | 0.6 |
|  |  | CFF8-2D8 | A | - | - | - | + | - | - | - | - | 47.7 |
|  |  | CFF8-2D9 | B1 | - | - | + | ND^3^ | - | *cnf2* | *cdtIII* | - | 1.1 |
|  |  | CFF8-2D10 | A | - | - | - | - | - | - | - | - | 41.9 |
|  |  | CFF8-2E2 | A | - | - | - | - | - | - | - | - | 0.2 |
| 14 | II | CFF14-1C12 | B2 | + | NA | + | NA | + | *cnf1* | - | - | NA |
|  |  | CFF14-1D2 | B2 | - | - | - | ND | + | - | - | - | 0.3 |
| 18 | III | CFF18-3D2 | D | - | - | - | - | - | - | - | - | 7.9 |
|  |  | CFF18-4B7 | A | - | - | - | ND | - | - | - | - | 1.8 |
|  |  | CFF18-3D5 | A | - | - | - | + | - | - | - | - | 39.7 |
| 31 | III | CFF31-6G11 | B2 | - | - | - | - | - | - | - | - | 1.7 |
|  |  | CFF31-6G2 | D | - | - | - | - | - | - | - | - | 0.2 |
|  |  | CFF31-6G9 | D | - | - | - | - | - | - | - | - | 0.6 |
|  |  | CFF31-6G8 | B2 | + | NA | + | NA | + | *cnf1* | - | - | NA |
|  |  | CFF31-6G10 | B2 | - | - | + | ND | - | - | *cdtIV* | - | 2.8 |
| 32 | I | CFF32-6A8 | B2 | + | NA | + | NA | + | *cnf1* | - | - | NA |
| 53 | II | CFF53-8A1 | D | - | - | - | + | - | - | - | - | 5.8 |
|  |  | CFF53-8A9 | B2 | + | NA | - | NA | + | - | - | - | NA |
|  |  | CFF53-8A10 | B2 | - | - | - | - | - | - | - | - | 0.6 |
| 68 | III | CFF68-10E8 | D | - | - | - | + | - | - | - | - | 0.6 |
|  |  | CFF68-10E9 | B2 | + | NA | + | NA | + | *cnf1* | - | - | NA |
| 117 | III | CFF117-17G2 | D | - | - | - | + | - | - | - | - | 48.8 |
|  |  | CFF117-17G3 | B2 | + | NA | + | NA | - | *cnf1* | - | - | NA |
|  |  | CFF117-17G7 | B1 | - | - | - | - | - | - | - | - | 1.9 |
| 142 | II | CFF142-18B1 | A | - | - | - | - | - | - | - | - | 0.8 |
| 150 | I | CFF150-19A4 | D | - | - | - | - | - | - | - | - | 3.6 |
| 158 | III | CFF158-19G1 | B2 | - | + | - | ND | + | - | - | - | 0.2 |
| 159 | IV | CFF159-19H2 | B2 | + | NA | + | NA | + | *cnf1* | *cdtIV* | - | NA |
|  |  | CFF159-19H4 | B1 | - | - | - | - | - | - | - | - | 0.9 |
| 162 | II | CFF162-20B4 | D | - | - | - | - | - | - | - | - | 29.0 |
|  |  | CFF162-20B6 | B2 | + | NA | + | NA | + | *cnf1* | - | - | NA |
| 163 | III | CFF163-20C1 | A | - | + | - | ND | - | - | - | + | 0.6 |
|  |  | CFF163-20C3 | B2 | - | + | + | ND | + | - | *cdtI* | - | 0.5 |
| 164 | II | CFF164-20D5 | B2 | + | NA | - | NA | + | - | - | - | NA |

^1,^ TNM (Tumor, lymph Nodes, Metastasis) staging system according to the [International Union Against Cancer](http://en.wikipedia.org/wiki/International_Union_Against_Cancer); ^2,^ Not applicable due to the presence of hemolysin inducing cell death ; ^3,^ Not determined because the strain harbored CM-encoding gene(s).
