# Supplementary material for: High Prevalence of Mucosa-Associated E. coli Producing Cyclomodulin and Genotoxin in Colon Cancer
Source: PLoS One. 2013 Feb 14;8(2):e56964. doi: 10.1371/journal.pone.0056964 (PMC3572998; doi:10.1371/journal.pone.0056964)
Supplement: Table S2 — E. coli strains isolated from proximal colon cancers. (DOCX) [file pone.0056964.s002.docx]

**Table S2. *E. coli* strains isolated from proximal colon cancers.**

| Patient | TNM  Stage^1^ | *E. coli* Strain | Phylogroup | Cytopathic effect | | | | CM-encoding gene | | | | Number of adherent  bacteria per cell |
| --- | --- | --- | --- | --- | --- | --- | --- | --- | --- | --- | --- | --- |
|  |  |  |  | Hly | pks-cif | cdt-cnf | *comet* | *pks* | *cnf* | *cdt* | *cif* |  |
| 15 | II | CFF15-3C4 | B1 | - | - | - | - | - | - | - | - | 0.2 |
|  |  | CFF15-4A9 | B2 | - | - | - | - | - | - | - | - | 0.2 |
| 16 | I | CFF16-2F8 | B2 | + | NA^2^ | + | NA^2^ | + | *cnf1* | *cdtIV* | - | NA^2^ |
|  |  | CFF16-2G3 | B1 | - | - | - | + | - | - | - | - | 0.1 |
|  |  | CFF16-2G5 | A | - | - | - | - | - | - | - | - | 0.1 |
| 37 | III | CFF37-2G2 | B2 | + | NA | - | NA | - | - | - | - | NA |
| 43 | II | CFF43-6B1 | B1 | - | - | - | - | - | - | - | - | 3.3 |
| 44 | I | CFF44-6C12 | A | - | - | - | - | - | - | - | - | 0.1 |
|  |  | CFF44-6C10 | A | - | - | - | - | - | - | - | - | 1.9 |
| 56 | II | CFF56-8F1 | B2 | + | NA | + | NA | - | *cnf1* | - | - | NA |
|  |  | CFF56-8F8 | A | - | - | - | - | - | - | - | - | 39.3 |
| 62 | II | CFF62-10C5 | A | - | - | + | ND^3^ | - | - | *cdtIV* | - | 0.2 |
|  |  | CFF62-10D8 | D | + | NA | - | NA | - | - | - | - | NA |
|  |  | CFF62-10D12 | B2 | - | + | - | ND | + | - | - | - | 0.4 |
| 82 | III | CFF82-11F1 | B2 | - | - | - | + | - | - | - | - | 4.5 |
| 83 | IV | CFF83-11G5 | B2 | - | + | - | ND | + | - | - | - | 3.0 |
| 85 | III | CFF85-11H5 | D | - | - | - | - | - | - | - | - | 9.0 |
| 87 | II | CFF87-12B1 | B2 | + | NA | + | NA | + | *cnf1* | - | - | NA |
| 107 | III | CFF107-13H2 | B2 | + | NA | + | NA | + | *cnf1* | - | - | NA |
|  |  | CFF107-13H4 | B1 | + | NA | - | NA | - | - | - | - | NA |
| 119 | IV | CFF119-14G4 | D | - | - | - | - | - | - | - | - | 0.7 |
| 120 | I | CFF120-14H2 | B1 | - | - | - | - | - | - | - | - | 43.5 |
|  |  | CFF120-14H4 | B2 | - | - | - | ND | + | - | - | - | 25.8 |
| 130 | I | CFF130-16C1 | B2 | - | + | - | ND | + | - | - | - | 2.0 |
| 138 | II | CFF138-18C3 | B2 | + | NA | + | NA | + | *cnf1* | - | - | NA |
|  |  | CFF138-18C5 | B2 | - | - | - | - | - | - | - | - | 2.2 |
| 144 | III | None |  |  |  |  |  |  |  |  |  |  |
| 149 | II | CFF149-18H5 | B2 | - | + | - | ND | + | - | - | - | 0.2 |
| 151 | IV | CFF151-19B8 | A | - | + | - | ND | - | - | - | + | 33.8 |
|  |  | CFF151-19C3 | A | - | - | - | - | - | - | - | - | 1.7 |
| 152 | II | CFF152-19D1 | B1 | - | + | - | ND | - | - | - | + | 1.7 |
|  |  | CFF152-19D12 | B2 | + | NA | + | NA | + | *cnf1* | - | - | NA |
| 153 | IV | CF153-19E5 | A | - | - | - | + | - | - | - | - | 7.7 |

^1,^ TNM (Tumor, lymph Nodes, Metastasis) staging system according to the [International Union Against Cancer](http://en.wikipedia.org/wiki/International_Union_Against_Cancer); ^2,^ Not applicable due to the presence of hemolysin inducing cell death ; ^3^, Not determined because the strain harbored CM-encoding gene(s).
