# Supplementary material for: High Prevalence of Mucosa-Associated E. coli Producing Cyclomodulin and Genotoxin in Colon Cancer
Source: PLoS One. 2013 Feb 14;8(2):e56964. doi: 10.1371/journal.pone.0056964 (PMC3572998; doi:10.1371/journal.pone.0056964)
Supplement: Table S3 — E. coli strains isolated from diverticulosis. (DOCX) [file pone.0056964.s003.docx]

**Table S3. *E. coli* strains isolated from diverticulosis.**

| Patient | *E. coli* Strain | Phylogroup | Cytopathic effect | | | | CM-encoding gene | | | | Number of adherent  bacteria per cell |
| --- | --- | --- | --- | --- | --- | --- | --- | --- | --- | --- | --- |
|  |  |  | Hly | pks-cif | cdt-cnf | Comet | *pks* | *cnf* | *cdt* | *cif* |  |
| 5 | CFF5-3B1 | A | - | - | - | + | - | - | - | - | 7.5 |
|  | CFF5-3B3 | D | - | - | - | - | - | - | - | - | 3.4 |
| 13 | CFF13-1A9 | D | - | - | - | + | - | - | - | - | 7.6 |
| 17 | None |  |  |  |  |  |  |  |  |  |  |
| 22 | CFF22-1D5 | B2 | - | - | - | - | - | - | - | - | 0.5 |
| 29 | None |  |  |  |  |  |  |  |  |  |  |
| 30 | CFF30-3F4 | A | - | - | - | + | - | - | - | - | 5.0 |
|  | CFF30-3E12 | D | - | - | - | + | - | - | - | - | 43.0 |
| 42 | CFF42-4D5 | B2 | + | NA^1^ | + | NA^1^ | + | *cnf1* |  | - | NA^1^ |
|  | CFF42-3F11 | A | - | - | - | + | - | - | - | - | 35.7 |
| 49 | CFF49-4E5 | D | - | - | - | - | - | - | - | - | 20.1 |
|  | CFF49-4E12 | A | - | - | - | - | - | - | - | - | 12.6 |
| 51 | CFF51-7E8 | A | - | - | - | - | - | - | - | - | 10.8 |
|  | CFF51-7E11 | D | - | - | - | - | - | - | - | - | 28.1 |
| 63 | CFF63-9D7 | B2 | - | - | - | - | - | - | - | - | 1.3 |
|  | CFF63-9D5 | D | - | - | - | - | - | - | - | - | 73.2 |
| 65 | CFF65-9F1 | B2 | - | - | - | - | - | - | - | - | 2.4 |
|  | CFF65-9F4 | B2 | - | - | - | - | - | - | - | - | 8.4 |
| 67 | CFF67-9H1 | D | - | - | - | + | - | - | - | - | 0.3 |
|  | CFF67-9H5 | D | - | - | - | - | - | - | - | - | 53.5 |
| 73 | CFF73-12H1 | B2 | - | - | - | ND^2^ | + | - | - | - | 0.1 |
|  | CFF73-12H2 | A | - | - | - | - | - | - | - | - | 9.3 |
|  | CFF73-12H11 | D | - | - | - | - | - | - | - | - | 29.8 |
| 74 | None |  |  |  |  |  |  |  |  |  |  |
| 77 | CFF77-11C4 | A | - | - | - | + | - | - | - | - | 59.8 |
| 79 | CFF79-11D3 | D | - | - | - | - | - |  |  | - | 28.0 |
|  | CFF79-11D9 | B2 | + | NA | + | NA | + | *cnf1* | - | - | NA |
| 96 | CFF96-18E2 | B1 | - | - | - | + | - | - | - | - | 20.7 |
|  | CFF96-18E6 | B2 | + | NA | - | NA | + | *cnf1* | - | - | NA |
| 99 | CFF99-13D1 | B2 | + | NA | + | NA | + | *cnf1* | - | - | NA |
|  | CFF99-13D5 | A | - | - | - | - | - | - | - | - | 21.2 |
| 103 | CFF103-13E3 | A | - | - | - | + | - | - | - | - | 0.6 |
|  | CFF103-13E6 | A | - | - | - | - | - | - | - | - | 4.6 |
|  | CFF103-13E9 | A | - | - | - | - | - | - | - | - | 9.7 |
|  | CFF103-13E11 | A | - | - | - | - | - | - | - | - | 8.6 |
| 109 | None |  |  |  |  |  |  |  |  |  |  |
| 110 | CFF110-17C1 | B2 | - | - | - | - | - | - | - | - | 0.1 |
|  | CFF110-17C7 | A | - | - | - | - | - | - | - | - | 0.4 |
| 112 | CFF112-17F2 | B2 | - | - | - | - | - | - | - | - | 3.9 |
| 113 | CFF113-17E1 | B2 | - | - | - | - | - | - | - | - | 0.1 |
| 116 | None |  |  |  |  |  |  |  |  |  |  |
| 121 | CFF124-15C1 | B2 | - | - | - | + | - | - | - | - | 4.2 |
| 124 | CFF124-15F5 | D | - | - | - | - | - | - | - | - | 0.2 |
|  | CFF124-16G2 | B1 | - | - | - | - | - | - | - | - | 0.9 |
|  | CFF124-17H1 | B1 | + | - | - | - | - | - | - | - | 0.4 |
| 125 | None |  |  |  |  |  |  |  |  |  |  |
| 126 | CFF126-15H2 | A | - | - | - | - | - | - | - | - | 9.4 |
| 127 | CFF127-16A4 | B2 | - | + | - | ND | + | - | - | - | 0.1 |
|  | CFF127-16A8 | B2 | - | - | - | - | - | - | - | - | 9.2 |
| 147 | CFF147-18G3 | A | - | - | - | + | - | - | - | - | 0.5 |
|  | CFF147-18G9 | A | - | - | - | + | - | - | - | - | 13.7 |
| 154 | CFF154-20E2 | A | - | - | - | + | - | - | - | - | 0.1 |

^1,^ Not applicable due to the presence of hemolysin inducing cell death ; ^2,^ Not determined because the strain harbored CM-encoding gene(s).
