# Supplementary material for: High Prevalence of Mucosa-Associated E. coli Producing Cyclomodulin and Genotoxin in Colon Cancer
Source: PLoS One. 2013 Feb 14;8(2):e56964. doi: 10.1371/journal.pone.0056964 (PMC3572998; doi:10.1371/journal.pone.0056964)
Supplement: Table S4 — Primers used in this study. (DOCX) [file pone.0056964.s004.docx]

**Table S4. Primers used in this study.**

| Primers | Oligonucleotide sequences 5’-3’(Tm of PCR) | Specificity | PCR product size (bp) | Reference |
| --- | --- | --- | --- | --- |
| pksORF9-10.1KJ | ATTCGATAGCGTCACCCAAC (58°C) | *clbK-clbJ* | 2 119 | [1] |
| pksORF9-10.2KJ | TAAGCGTCTGGAATGCAGTG |  |  |  |
| IHAPJPN42 | CAGATACACAGATACCATTCA (55°C) | *clbA* | 1002 | [2] |
| IHAPJPN46 | CTAGATTATCCGTGGCGATTC |  |  |  |
| IHAPJPN55 | TTATCCTGTTAGCTTTCGTTC (55°C) | *clbQ* | 821 | [2] |
| IHAPJPN56 | CTTGTATAGTTACACAACTATTTC |  |  |  |
| CNF-1s | GGGGGAAGTACAGAAGAATTA (48°C) | *cnf1* | 1112 | [3] |
| CNF-1as | TTGCCGTCCACTCTCACCAGT |  |  |  |
| CNF-2s | TATCATACGGCAGGAGGAAGCACC (48°C) | *cnf2* | 1241 | [4] |
| CNF-2as | GTCACAATAGACAATAATTTTCCG |  |  |  |
| CNF3-3D | TAACGTAATTAGCAAAGA (48°C) | *cnf3* | 757 | [5,6] |
| CNF-3as | GTCTTCATTACTTACAGT |  |  |  |
| CDT-s1 | GAAAGTAAATGGAATATAAATGTCCG (60°C) | *cdtB-II, cdtB-III, cdtB-V* | 467 | [3] |
| CDT-as1 | AAATCACCAAGAATCATCCAGTTA |  |  |  |
| CDT-IIas ^a^ | TTTGTGTTGCCGCCGCTGGTGAAA (62°C) | *cdtB-II* | 556 | [3] |
| CDT-IIIas ^a^ | TTTGTGTCGGTGCAGCAGGGAAAA (62°C) | *cdtB-III, cdtB-V* | 555 | [3] |
| CDT-s2 | GAAAATAAATGGAACACACATGTCCG (56°C) | *cdtB-I, cdtB-IV* | 467 | [3] |
| CDT-as2 | AAATCTCCTGCAATCATCCAGTTA |  |  |  |
| CDT-Is | CAATAGTCGCCCACAGGA (56°C) | *cdtB-I* | 411 | [3] |
| CDT-Ias | ATAATCAAGAACACCACCAC |  |  |  |
| CDT-IVs | CCTGATGGTTCAGGAGGCTGGTTC (56°C) | *cdtB-IV* | 350 | [3] |
| CDT-IVas | TTGCTCCAGAATCTATACCT |  |  |  |
| P105 | GTCAACGAACATTAGATTAT (49°C) | *cdtC-V* | 748 | [7] |
| c2767r | ATGGTCATGCTTTGTTATAT |  |  |  |
| cif-int-s | AACAGATGGCAACAGACTGG (55°C) | *cif* | 383 | [8] |
| cif-int-as | AGTCAATGCTTTATGCGTCAT |  |  |  |

^a^ used with CDT-s1 primer.

**References**

1. Nougayrède J-P, Homburg S, Taieb F, Boury M, Brzuszkiewicz E, et al. (2006) Escherichia coli induces DNA double-strand breaks in eukaryotic cells. Science 313: 848–851. doi:10.1126/science.1127059.

2. Johnson JR, Johnston B, Kuskowski MA, Nougayrede J-P, Oswald E (2008) Molecular epidemiology and phylogenetic distribution of the Escherichia coli pks genomic island. J Clin Microbiol 46: 3906–3911. doi:10.1128/JCM.00949-08.

3. Tóth I, Hérault F, Beutin L, Oswald E (2003) Production of cytolethal distending toxins by pathogenic Escherichia coli strains isolated from human and animal sources: establishment of the existence of a new cdt variant (Type IV). J Clin Microbiol 41: 4285–4291.

4. Van Bost S, Jacquemin E, Oswald E, Mainil J (2003) Multiplex PCRs for identification of necrotoxigenic Escherichia coli. J Clin Microbiol 41: 4480–4482.

5. Orden JA, Domínguez-Bernal G, Martínez-Pulgarín S, Blanco M, Blanco JE, et al. (2007) Necrotoxigenic Escherichia coli from sheep and goats produce a new type of cytotoxic necrotizing factor (CNF3) associated with the eae and ehxA genes. Int Microbiol 10: 47–55.

6. Dubois D, Delmas J, Cady A, Robin F, Sivignon A, et al. (2010) Cyclomodulins in urosepsis strains of Escherichia coli. J Clin Microbiol 48: 2122–2129. doi:10.1128/JCM.02365-09.

7. Janka A, Bielaszewska M, Dobrindt U, Greune L, Schmidt MA, et al. (2003) Cytolethal distending toxin gene cluster in enterohemorrhagic Escherichia coli O157:H- and O157:H7: characterization and evolutionary considerations. Infect Immun 71: 3634–3638.

8. Marchès O, Ledger TN, Boury M, Ohara M, Tu X, et al. (2003) Enteropathogenic and enterohaemorrhagic Escherichia coli deliver a novel effector called Cif, which blocks cell cycle G2/M transition. Mol Microbiol 50: 1553–1567.
