# Supplementary material for: High Prevalence of Mucosa-Associated E. coli Producing Cyclomodulin and Genotoxin in Colon Cancer
Source: PLoS One. 2013 Feb 14;8(2):e56964. doi: 10.1371/journal.pone.0056964 (PMC3572998; doi:10.1371/journal.pone.0056964)
Supplement: Table S5 — Archetypal E. coli control strains used in this study. (DOCX) [file pone.0056964.s005.docx]

Table S5. Archetypal *E. coli* control strains used in this study.

| E. coli strains | Genes | Host origin | Reference |
| --- | --- | --- | --- |
| 28C | *hly, cnf1, cdt-IV, clbA*^a^*, clbK*^a^*, clbJ*^a^*, clbQ*^a^ | Porcine septicemia | [1] |
| 1404 | *cnf2, cdt-III* | Bovine septicemia | [2] |
| IHE3034 Δ*clbP* | *cdt-I, clb* Δ*clbP* | Newborn meningitis | [3] |
| DH5α pCP2123 | *cdt-II* | Laboratory strain | [4] |
| C48a | *cnf3, eae, cif*^a^ | Healthy lamb | [5] |
| DH10β pBAC*pks* | *clb* | Laboratory strain | [3] |
| E22 | *cif, eae* | Rabbit - EPEC | [6] |
| RS218 | PCR-based phylotyping | Newborn meningitis | [7] |
| EDL933 | *eae, stx1, stx2* | EHEC | [8] |
| DH5α |  | Laboratory strain | Novagen |

^a^ genes detected during this study.

**References**

1. Dozois CM, Clément S, Desautels C, Oswald E, Fairbrother JM (1997) Expression of P, S, and F1C adhesins by cytotoxic necrotizing factor 1-producing Escherichia coli from septicemic and diarrheic pigs. FEMS Microbiol Lett 152: 307–312.

2. Oswald E, de Rycke J, Lintermans P, van Muylem K, Mainil J, et al. (1991) Virulence factors associated with cytotoxic necrotizing factor type two in bovine diarrheic and septicemic strains of Escherichia coli. J Clin Microbiol 29: 2522–2527.

3. Nougayrède J-P, Homburg S, Taieb F, Boury M, Brzuszkiewicz E, et al. (2006) Escherichia coli induces DNA double-strand breaks in eukaryotic cells. Science 313: 848–851. doi:10.1126/science.1127059.

4. Pickett CL, Cottle DL, Pesci EC, Bikah G (1994) Cloning, sequencing, and expression of the Escherichia coli cytolethal distending toxin genes. Infect Immun 62: 1046–1051.

5. Orden JA, Domínguez-Bernal G, Martínez-Pulgarín S, Blanco M, Blanco JE, et al. (2007) Necrotoxigenic Escherichia coli from sheep and goats produce a new type of cytotoxic necrotizing factor (CNF3) associated with the eae and ehxA genes. Int Microbiol 10: 47–55.

6. Marchès O, Ledger TN, Boury M, Ohara M, Tu X, et al. (2003) Enteropathogenic and enterohaemorrhagic Escherichia coli deliver a novel effector called Cif, which blocks cell cycle G2/M transition. Mol Microbiol 50: 1553–1567.

7. Silver RP, Aaronson W, Sutton A, Schneerson R (1980) Comparative analysis of plasmids and some metabolic characteristics of Escherichia coli K1 from diseased and healthy individuals. Infect Immun 29: 200–206.

8. Perna NT, Plunkett G 3rd, Burland V, Mau B, Glasner JD, et al. (2001) Genome sequence of enterohaemorrhagic Escherichia coli O157:H7. Nature 409: 529–533. doi:10.1038/35054089.
